# HDF1, a novel flowering time regulator identified in a mutant suppressing *sensitivity to red light reduced 1* early flowering

**DOI:** 10.1038/s41598-023-28049-6

**Published:** 2023-01-25

**Authors:** Mikael Johansson, Alexander Steffen, Martin Lewinski, Natalie Kobi, Dorothee Staiger

**Affiliations:** RNA Biology and Molecular Physiology, Bielefeld University, Universitaetsstrasse 25, 33615 Bielefeld, Germany

**Keywords:** Next-generation sequencing, Gene expression, Flowering, Plant molecular biology

## Abstract

Arabidopsis SENSITIVITY TO RED LIGHT REDUCED 1 (SRR1) delays the transition from vegetative to reproductive development in noninductive conditions. A second-site suppressor screen for novel genes that overcome early flowering of *srr1-1* identified a range of *suppressor of srr1-1 mutants* flowering later than *srr1-1* in short photoperiods. Here, we focus on mutants flowering with leaf numbers intermediate between *srr1-1* and Col*. Ssm67* overcomes *srr1-1* early flowering independently of day-length and ambient temperature. Full-genome sequencing and linkage mapping identified a causative SNP in a gene encoding a Haloacid dehalogenase superfamily protein, named HAD-FAMILY REGULATOR OF DEVELOPMENT AND FLOWERING 1 (HDF1). Both, *ssm67* and *hdf1-1* show increased levels of *FLC*, indicating that HDF1 is a novel regulator of this floral repressor. HDF1 regulates flowering largely independent of SRR1, as the effect is visible in *srr1-1* and in Col, but full activity on *FLC* may require SRR1. Furthermore, *srr1-1* has a delayed leaf initiation rate that is dependent on HDF1, suggesting that SRR1 and HDF1 act together in leaf initiation. Another mutant flowering intermediate between *srr1-1* and wt, *ssm15*, was identified as a new allele of ARABIDOPSIS SUMO PROTEASE 1, previously implicated in the regulation of FLC stability.

## Introduction

Higher plants carefully time the transition to flowering, allowing for maximal acquisition of biomass during the growth season to enhance reproductive success. Environmental factors such as day length and temperature are integrated with endogenous developmental processes, creating a network of signaling pathways to find the optimal time for flowering^1,2^.

In the facultative long day (LD) plant *Arabidopsis thaliana*, a long daily light period promotes flowering via the photoperiodic pathway^3,4^. Day length is sensed in the leaves by photoreceptors and integrated into the flowering time network via the circadian clock. The key regulator of the photoperiodic pathway is CONSTANS (CO) and the oscillatory pattern of the *CO* transcript is in large parts shaped by the repressive action of CYCLING DOF FACTORS (CDFs) which prevent *CO* accumulation in the morning^5,6^. Instead, CO protein accumulates in the evening and activates the floral integrator *FLOWERING LOCUS T* (*FT*). The FT protein moves from leaves to the shoot apical meristem (SAM) to induce flowering^7–9^. Important antagonists of CO are TEMPRANILLO (TEM) transcription factors which repress expression of *FT*, thereby counteracting the effect of CO and balancing the floral response to LDs^10^.

The phytohormone gibberellin (GA) has a well-established function in the control of flowering^11^. GA is required for flowering in short days (SDs)^12^ as GA accumulation in the SAM activates *LEAFY* and *SUPPRESSOR OF OVEREXPRESSION OF CONSTANS 1* (*SOC1*) which enables flowering without a photoperiodic stimulus^13,14^. In addition, GA has spatially separated functions in LDs, by promoting expression of *FT* in the vascular tissue and activation of SQUAMOSA PROMOTER BINDING PROTEIN-LIKE (SPL) transcription factors in the meristem^15^.

Vernalization, the exposure to an extended period of cold, promotes flowering by down-regulating the MADS-box transcription factor FLOWERING LOCUS C (FLC)^16,17^. *FLC* is also down-regulated by endogenous regulators, collectively referred to as the autonomous pathway (AP) which comprises a suite of RNA binding proteins and chromatin modifiers^18–20^. FLC negatively regulates a set of floral activators, among them FT^21^. It has evolved as a central regulatory hub for flowering time in the *Brassicaceae* and the mechanisms of *FLC* transcriptional regulation and epigenetic silencing are extensively studied (reviewed in^22^). Additionally, *FLC* expression might also be regulated indirectly by phosphorylation of AP components through Casein Kinase II and either upregulated or repressed through Protein Phosphatase 2A dephosphorylation, depending on the subunit involved^23,24^.

Apart from extended periods of low temperature, ambient temperature also impacts flowering, with increasing temperature accelerating floral transition. Mutants deficient in the photoreceptor PhyB flower earlier than wild type (wt) plants at 22 °C but not at 16 °C, indicating that PhyB is involved in temperature perception^25^.

We have previously characterized the role of SENSITIVITY TO RED LIGHT REDUCED (SRR1) in flowering time control. SRR1 was originally identified as a component of phyB signaling due to the reduced sensitivity to red light in *srr1-1*^26^. *Srr1-1* mutant plants also have a reduced ability to sense the length of the photoperiod, leading to particularly early flowering in SDs^27^. By promoting the expression of direct repressors of *FT* including *CDF1* and the *TEM* transcription factors, SRR1 represses flowering in non-inductive conditions^27^. However, SRR1 function is not limited to the photoperiodic pathway. Levels of the key floral repressor *FLC* are also repressed in the *srr1-1* mutant. In other *Brassicaceae*, *Brassica rapa BrFLC2* was identified as a candidate for a major flowering QTL co-regulating a *cis*-QTL containing *BrSRR1*, thus associating *BrSRR1* with flowering time control^28^. Moreover, *Brassica napus* harbours five *SRR1* homologs which show subfunctionalization^29^. Recently, SNPs in the SRR1 orthologue of *Brassica juncea* were shown to be associated with flowering time also in this species^30^.

SRR1 is a pioneer protein with hitherto uncharacterized biochemical properties. To begin to understand the molecular underpinnings of its action in flowering time control we embarked on a second-site suppressor screen in Arabidopsis. A screen of an ethylmethanesulfonate (EMS) mutagenized *srr1-1* population revealed several mutants that partially rescued the early flowering of *srr1-*1.

Here, we identify two of the *suppressor of srr1-1 mutants* (*ssm*), *ssm15* and *ssm67*, which suppress *srr1-1* early flowering independent of temperature and day-length. Full genome resequencing combined with a classical mapping approach identified the causal mutation in *ssm15* as a G to A single nucleotide polymorphism (SNP) in exon 6 of ARABIDOPSIS SUMO PROTEASE 1 (ASP1), a protein already known to regulate FLC protein stability^31^. *Ssm67* revealed a C to T transition in the At5g10460 gene encoding a Haloacid dehalogenase (HAD) superfamily protein. HADs represent an evolutionary ancient group of enzymes with diverse functions^32^ but have not been implicated in flowering time regulation so far. Besides the name-giving haloacid dehalogenases that cleave carbon-halogen bonds and are mostly present in prokaryotes, the superfamily comprises ATPases, phosphoesterases, phosphonatases and sugar phosphomutases acting on a wide variety of substrates. Their core catalytic domain consists of a Rossmannoid-fold, a distinct structure of alternating β-strands and α-helices which encompasses the active sites characterized by four diagnostic motifs. Motif I contains two Asp residues (DxD), catalysing a nucleophilic attack on the substrate. Motif II and III contain a conserved Thr, Ser or Lys residue and motif IV is comprised of the sequences DD, GDxxxD, or GDxxxxD. Many HADs also contain capping domains with a high degree of sequence variation that shield the catalytic site. Based on their size and their structural properties, they are defined as either C0, C1, or C2 caps. Together with the catalytic motifs, they determine the substrate specificity of the enzyme^33^. Although there are HADs that act on proteins as their substrates, the vast majority targets small molecules whose identities cannot be deduced from the sequence of the catalytic sites but have to be determined experimentally^32–34^. In Arabidopsis, about 200 genes encoding HAD enzymes were identified, but to date only few have been characterized^35–37^. Our investigation revealed that the mutation in the HAD responsible for the *ssm67* phenotype acts to reduce levels of the floral repressor *FLC*. This is the first time that HADs could be assigned a role in flowering time control.

## Results

### A second site suppressor screen of the *srr1-1* mutant

*Arabidopsis thaliana* SRR1 delays the transition to flowering by indirectly repressing the expression of the floral integrator *FT* under noninductive growth conditions^27^. In a search for novel genes important for the regulation of flowering by SRR1, a second site suppressor screen of the *srr1-1* loss-of-function mutant was performed. The *srr1-1* mutant flowers particularly early in SDs. As loss of SRR1 and PhyB additively accelerate flowering time at ambient temperatures, the screen was performed at 16 °C, where PhyB has no influence on flowering time^25^. *Srr1-1* seeds were EMS-mutagenized and for M2 plants the number of leaves at bolting was recorded.

**Figure 1:**
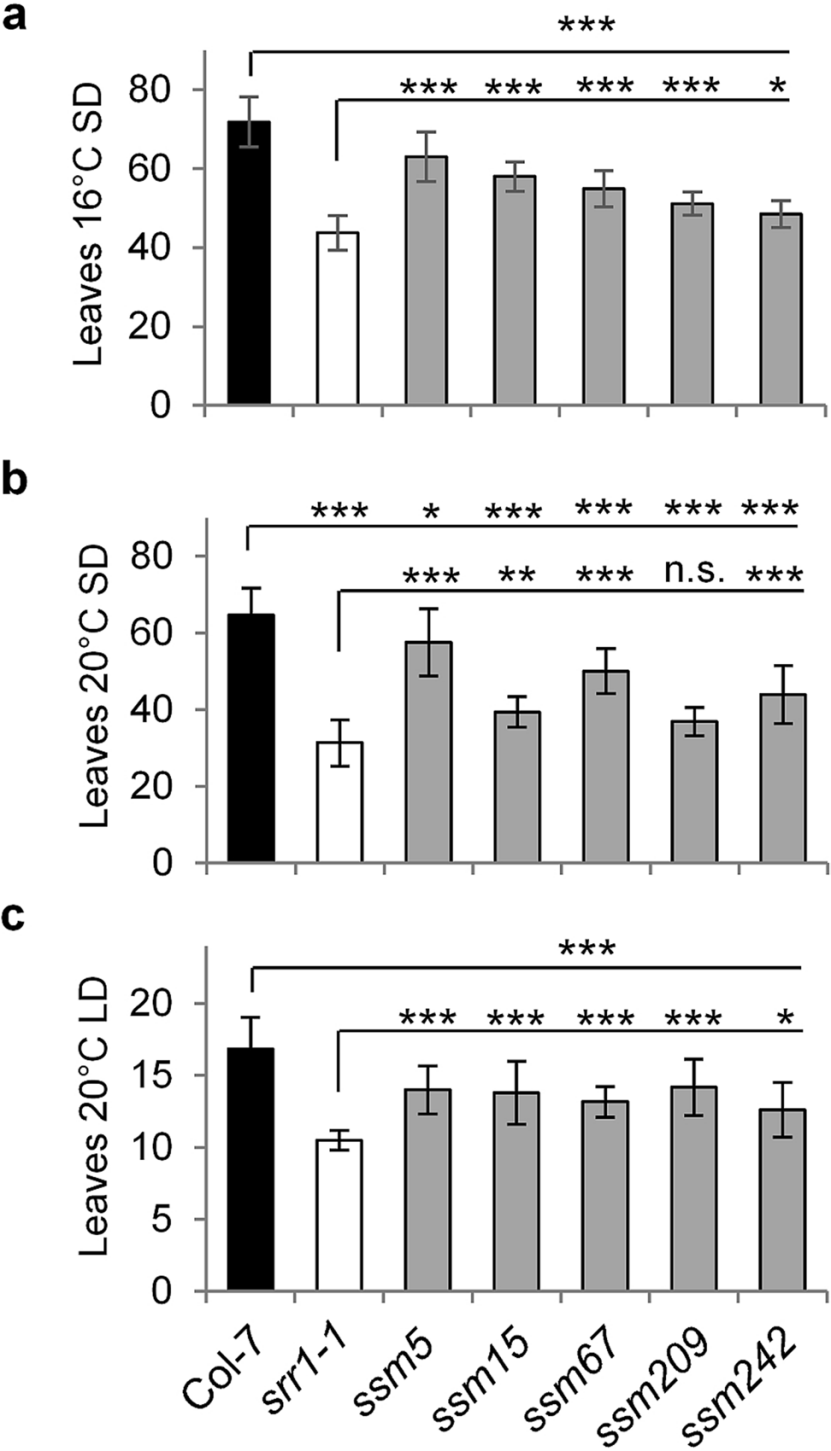
Flowering time of *suppressor of srr1-1* (*ssm*) candidate mutants. Candidate *ssm* mutants with an intermediary phenotype between Col-7 wt and *srr1-1* selected in an initial screen were rescreened in SDs at 16 °C (**a**), in SDs at 20 °C (**b**), and LDs at 20 °C (**c**). The number of leaves was counted for 10–12 plants each. Data are means ± s.d. To determine the statistical significance, an ANOVA followed by a post-hoc Dunnett-test was performed (*P ≤ 0.05, **P ≤ 0.01, ***P ≤ 0.001, *n.s.* not significant).

In the initial screen, we identified several *ssm* candidates which bolted significantly later than *srr1-1* and produced viable seeds. Here, we focus on candidate suppressors with a flowering phenotype intermediate between *srr1-1* and Col-7 wt, defined by a significant difference from *srr1-1* as well as from wt (Fig. 1a). As the initial screen was performed at 16 °C, the five *ssm* mutants *ssm5*, *ssm15*, *ssm67*, *ssm209*, and *ssm242* were rescreened at 20 °C in SDs to determine whether the flowering phenotype was temperature-dependent. The mutants again partially rescued early flowering of the *srr1-1* mutant, similarly as at 16 °C. The only exception was *ssm209* which did not flower significantly later than *srr1-1* and therefore showed a temperature-dependent suppression of the *srr1-1* flowering phenotype (Fig. 1b).

Similarly, to account for any photoperiodic effects, flowering time of the *ssm* mutants was also tested in 20 °C LD conditions. All plant lines flowered earlier than in SDs, which shows that recognition of day length via the photoperiodic pathway was not compromised. The five *ssm* mutants still were flowering intermediate between *srr1-1* and wt, therefore partially rescuing the early flowering of *srr1-1* independently of day length (Fig. 1c).

Here, we focus on the candidate *ssm67* which reproducibly produced the most robust phenotypes and flowered with a significant difference from *srr1-1* as well as from wt under all conditions tested (Fig. 1a–c). Additionally, as *ssm15* showed a strong intermediate phenotype especially at 16 °C SDs (Fig. 1a), we also identified the causal mutation of this mutant. *Ssm5* also showed robust phenotypes under all tested conditions, but the plants were extremely difficult to propagate and produced little viable seeds, therefore we had to exclude this mutant from further analysis.

### Identification of the *ssm15* mutation

To identify the causal single nucleotide polymorphism (SNP) of the *ssm15* mutant, a bulk segregant analysis using full genome sequencing was performed, as outlined in Suppl. Fig. S1^38^. Therefore, *ssm15* was back-crossed to *srr1-1*. F1 plants were selfed and the resulting segregating BC1F2 population was grown and scored for flowering time in SDs at 16 °C. Plants that displayed the suppressor phenotype were identified and material from each of these plants was pooled. The resulting DNA pool was submitted to full genome re-sequencing, with a coverage of ca. 60 ×. A pool of *srr1-1* plants was sequenced as a reference. The sequencing revealed 5845 SNPs different between the TAIR10 reference genome and the *srr1-1* or *ssm15* mutants, respectively. Of these, 771 SNPs were unique between *srr1-1* and *ssm15*. A SNP index was calculated to score the identified SNPs, leading to a number of candidate genes which were, however, distributed on chromosomes I, III, and IV (Suppl. Fig. S2a).

To asses which SNP was associated with the suppressor phenotype, a mapping population was created by crossing *ssm15* with the Landsberg *erecta* wt. Mapping with SSLP and dCAPS markers (Suppl. Table S1) revealed the lowest recombination frequency with the marker F21M12 on the upper arm of chromosome I (Suppl. Fig. S2b). For candidates on chromosome I in the full genome re-sequencing, we ultimately found a G to A transition in exon 6 of ARABIDOPSIS SUMO PROTEASE 1 (ASP1), leading to an Ala to Thr exchange (Suppl. Fig. S2c). ASP1 was recently shown to positively regulate the transition to flowering at least partly by repressing FLC protein stability^31^, confirming a role in flowering time control also revealed here in our suppressor screen. As ASP1 has already been characterized with respect to flowering, we decided to focus on *ssm67*.

### Identification of the *ssm67* mutation

**Figure 2:**
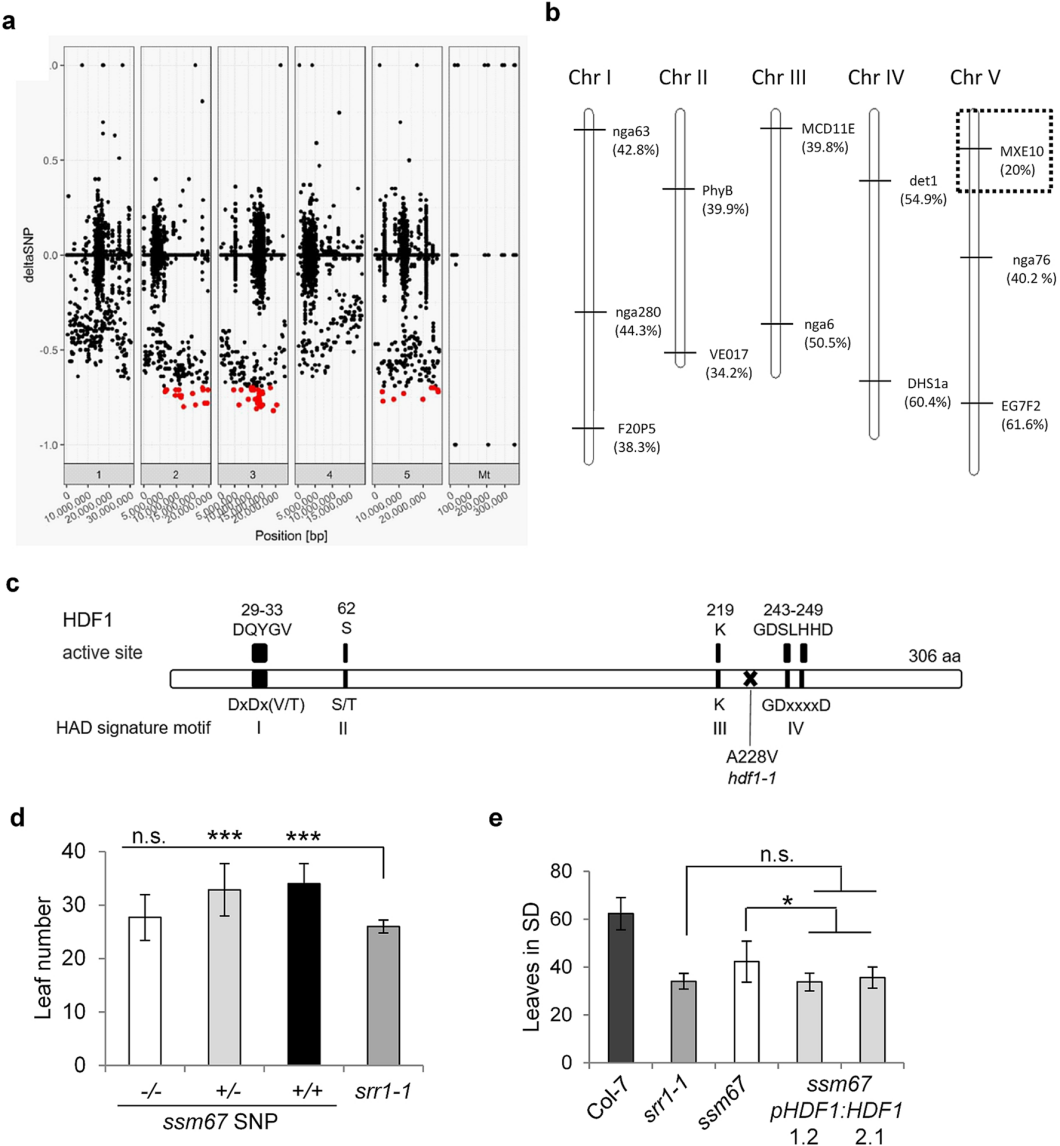
Identification of the causal mutation in *ssm67.* (**a**) Distribution of all identified SNPs in the *ssm67* genome and their SNP index compared to the *srr1-1* genome. A ΔSNP index closer to − 1.0 indicates SNPs unique for *ssm67*. The red dots denote SNPs ≤ − 0.7, all SNPs below this value were evaluated as potential causal SNPs. (**b**) Linkage mapping of the causal mutation in a *ssm67* × L. *erecta* mapping population using SSLP and CAPS markers. (**c**) Scheme of the At5g10460 Haloacid dehalogenase-like hydrolase (HAD). The position of the Ala to Val exchange is indicated. The consensus sequences of the HAD signature motifs I-IV are given below the boxes, the sequences of HDF1 are given above the boxes. (**d**) Flowering time of the segregating BC1F2 *ssm67* population. Leaf number was scored in LDs for 10–12 plants each. −/− homozygous wt, +/− heterozygous for causal mutation, +/+ homozygous for causal mutation. Data are means ± s.d. A *t* test was employed to determine statistical significance (***P ≤ 0.001, *n.s.* not significant). (**e**) Complementation of the *ssm67* late flowering phenotype by genomic *HDF1*. The leaf number of two independent lines homozygous for the genomic *HDF1* construct in the *ssm67* background, Col-7 wt plants, and the *srr1-1* and *ssm67* mutants was determined in SDs. For all lines, 15–20 plants were counted. Data are means ± s.d. A *t* test was employed to determine statistical significance. (*P ≤ 0.05, *n.s.* not significant).

To identify the causal SNP of the *ssm67* mutant, a bulk segregant analysis using full genome sequencing was performed, as outlined before (Suppl. Fig. S1). F1 plants of the backcross of *ssm67* to *srr1-1* were selfed and the resulting segregating BC1F2 population was scored for flowering time in SDs at 16 °C. Sequencing of pooled plants with the suppressor phenotype revealed 5500 SNPs different between the TAIR10 reference genome and the *srr1-1* or *ssm67* mutants, respectively, with 739 SNPs unique between *srr1-1* and *ssm67*. Calculation of the SNP index revealed a number of candidate loci on each of the chromosomes II, III, and V (Fig. 2a).

To map the *ssm67* locus, a linkage mapping population was created by crossing the *ssm67* mutant with *L*. *erecta* and screened for plants with the late-flowering suppressor phenotype. A rough mapping localized the *ssm67* locus to the top of chromosome V (Fig. 2b). This was confirmed by fine-scale mapping with the markers F15M7 and T31P16.

In the corresponding region on the top of chromosome V, three SNP candidates were located that caused nonsynonymous amino acid changes (Suppl. Table S2). To distinguish between these mutant candidates, dCAPS markers for the SNP in each candidate gene were designed to genotype individuals from a *srr1-1* × *ssm67* BC1F2 population with a stable *ssm67* phenotype (Suppl. Table S1). Only one of the SNPs was present in all lines with a suppressor phenotype, namely a C to T transition in position 683 of the At5g10460 locus. This mutation was further confirmed by Sanger sequencing. The gene consists of seven exons and encodes a HAD superfamily protein of unknown function with a length of 306 amino acids. The mutation leads to an Alanine to Valine change in amino acid 228 (Fig. 2c).

Genotyping of the segregating BC1F2 *ssm67* population scored for its flowering phenotype revealed that plants either heterozygous or homozygous for the causal mutation flowered significantly later than *srr1-1*, confirming a dominant segregation of the *ssm67* mutation and explaining the SNP distribution pattern observed in the sequencing analysis (Fig. 2d).

To further test whether At5g10460 is the causal gene for the *ssm67* phenotype, a construct consisting of ca. 1.5 kb of the endogenous promoter and the genomic sequence of At5g10460 was introduced into *ssm67* using *Agrobacterium*-mediated transformation. The construct was able to revert the intermediate flowering phenotype of *ssm67* back to the early flowering of *srr1-1*, indicating that At5g10460 indeed is responsible for the *ssm67* late flowering phenotype (Fig. 2e). Based on the flowering phenotype and the overall appearance of the plants, we named this protein HAD-FAMILY REGULATOR OF DEVELOPMENT AND FLOWERING 1 (HDF1) and the identified allele *hdf1-1*. HADs represent an evolutionary ancient group of enzymes with diverse functions^32^. The active sites are characterized by four diagnostic motifs. Motif I contains two Asp residues (DxD), catalysing a nucleophilic attack on the substrate. Motif II and III contain a conserved Thr, Ser or Lys residue and motif IV is comprised of the sequences DD, GDxxxD, or GDxxxxD.

We performed a whole sequence alignment of 93 HAD superfamily proteins from a dataset compiled of previously identified HADs^35,37^ and our own search in the Araport 11 database. We found all four diagnostic motifs to be well conserved. However, HDF1 contains only the first of the two conserved Asp in motif I which in HAD phosphatases serves as a nucleophile attacking the phosphoryl group of the substrate. The second Asp is positioned two residues C-terminal of the first Asp and protonates the leaving group in the first part of the reaction and deprotonates a water molecule in the second part of the reaction^34^. In this position, HDF1 instead contains a Tyr. The absence of the second Asp in HDF1 raises the possibility that is does not act as a phosphatase. Instead, the presence of a Tyr in the Asp + 2 position is characteristic for HADs with dehalogenase function in bacteria^33^. Interestingly, we found six candidates in Arabidopsis also missing the Asp in the + 2 position, but none of them displays a Tyr.

**Figure 3:**
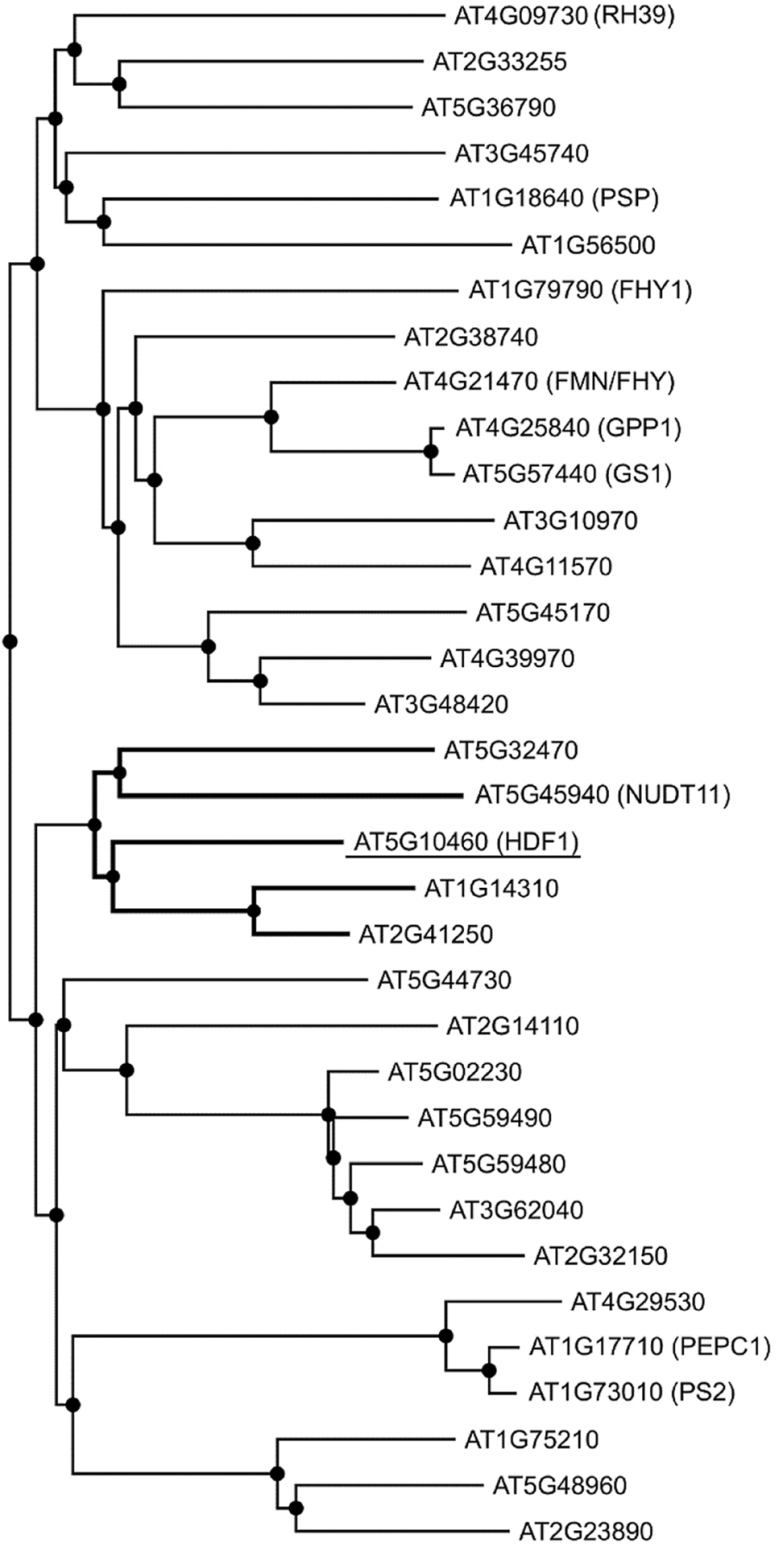
Phylogenetic analysis of HDF1*.* Phylogenetic tree of HAD superfamily proteins from Arabidopsis neighboring HDF1. Alignment of the predicted sequences was made using MAFFT version 7. The phylogenetic tree was constructed using the PHYML 3.0 web server. The complete tree can be seen in Suppl. Fig. S3.

Based on the alignment, we constructed a phylogenetic tree of all 93 HADs we previously identified (Suppl. Fig. S3a). From this tree, we further investigated the branch with the closest neighboring *HDF1* paralogs (Fig. 3). Due to the lack of other proteins with a Tyr in the + 2 position, HDF1 groups together with the uncharacterized HADs At1g14310 displaying a Gly in the + 2 position of motif I and At2g41250 displaying a Val residue in the + 2 position. In close vicinity we find the CoA phosphohydrolase NUDX11^39^ and the thiamin monophosphate phosphatase TH2^40^ (Suppl. Fig. S3b). Although HDF1 was the only HAD family protein with a Tyr in the + 2 position that we have found in the Col reference genome, it seems to be well conserved in other plant species, especially the *Brassicaceae,* with one copy in *Arabidopsis lyrata*, three copies in *Camelina sativa*, one copy each in *Brassica rapa* and *Brassica oleracea*, and two copies in *Brassica napus* (Suppl. Fig. S4). This points towards a very distinctive role of HDF1, as it apparently emerged early in the evolution of the *Brassicaceae* but has retained a very high degree of sequence conservation.

HADs are divided into several subclasses, based on the location of a so-called “capping domain”^41,42^. HDF1 belongs to the Class 2A, where the capping domain is found between the second and third motifs. The presence of the larger C2 cap shielding the active site suggests that the enzyme acts on a small molecule rather than on a protein. So far, the substrate remains elusive. HDF1 was identified in a search for sequences related to AtFHy1, a flavin mononucleotide (FMN) hydrolase involved in turnover of FMN that we also find distantly related to HDF1^35^. Subsequently, it was shown that recombinant AtFHy1 in vitro dephosphorylates 5-amino-6-ribitylamino-2,4(1H,3H) pyrimidinedione 5′-phosphate (ARPP), an intermediate of the riboflavin biosynthetic pathway, while recombinant HDF1 does not, excluding a role in riboflavin biosynthesis^43^.

### Impact of the *ssm67* mutant on development

Many mutants in genes regulating flowering time display additional pleiotropic phenotypes that affect development during the vegetative growth stages^44^. We previously found phenotypical differences between *srr1-1* and wt that we attributed mainly to a misregulation of PhyB-mediated red light signaling, showing that the function of SRR1 was not limited to control flowering time^26^. Therefore, we assayed how vegetative development was affected in *srr1-1* and *ssm67*.

**Figure 4:**
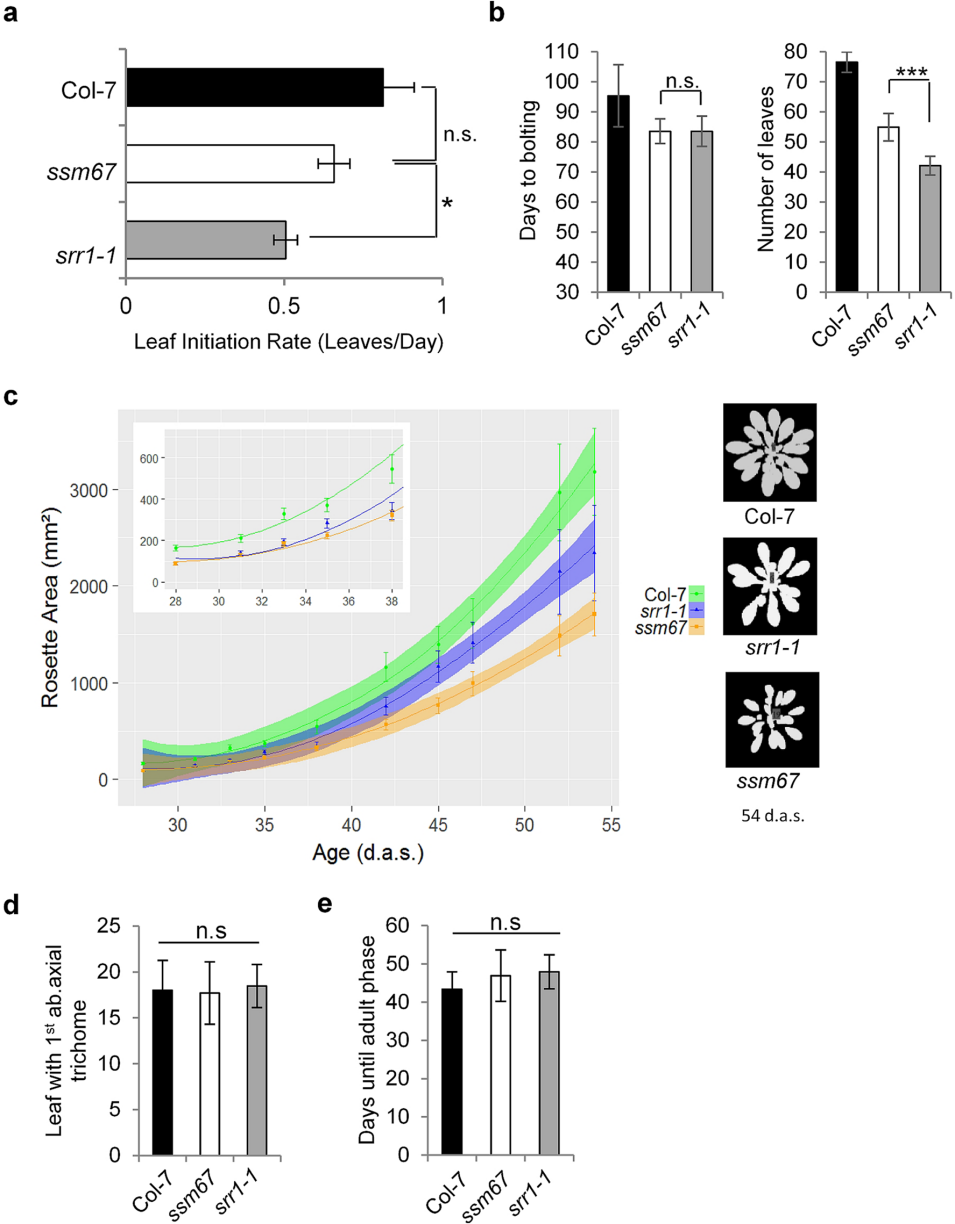
The *ssm67* mutation affects plant development. (**a**) Leaf initiation rate of *ssm67* compared to Col-7 wt and *srr1-1.* (**b**) Flowering time of *ssm67* compared to Col-7 wt and *srr1-1* in SDs, measured in days to bolting (left) and leaves to bolting (right) from n = 15–20 plants. Data are means ± s.d. To determine statistical significance, a Kruskal–Wallis test was performed (*P ≤ 0.05, ***P ≤ 0.001, *n.s.* not significant). (**c**) Rosette size of *ssm67* compared to Col-7 wt and *srr1-1* in SDs. (**d**,**e**) Trichome distribution. Col-7 wt, *srr1-1*, and *ssm67* mutant plants were grown in SDs at 20 °C. The leaf with the first abaxial trichome (**d**) and the number of days until first abaxial trichome (**e**) were observed (n = 15–20 plants, data are means ± s.d). To determine statistical significance, a Kruskal–Wallis test was performed (*n.s*. not significant).

A measure of plant development is the rate of emergence of new leaves. To examine whether the higher number of leaves at bolting in *ssm67* compared to *srr1-1* (Fig. 1) is due to changes in the leaf initiation rate, leaf numbers were counted every third day on plants grown in SDs. This revealed that *srr1-1* itself has a slower leaf initiation than wt, contributing to its lower leaf number at flowering (Fig. 4a). Thus, SRR1 in addition to its role in flowering likely has a role in vegetative development. The *ssm67* mutant had a statistically significant (p < 0.05) increase in leaf initiation rate compared to *srr1-1*, therefore partially rescuing also this phenotype of *srr1-1* (Fig. 4a). The average number of days to flowering was not significantly different between *srr1-1* and *ssm67*, but *ssm67* produced more leaves in the same time frame than *srr1-1* (Fig. 4b). Thus, HDF1 may act in the same molecular pathway as SRR1 to regulate leaf initiation.

To quantify general growth, rosette size was tracked in SD-grown plants from 30 days after germination until plants started to flower. This revealed that *srr1-1* plants themselves have a smaller rosette compared to wt, but also that the *hdf1-1* mutation in *ssm67* leads to a further decrease in size (Fig. 4c). This points towards an SRR1 independent effect of HDF1 on general plant growth, in addition to the changed leaf initiation rate.

To determine whether in addition to the adult-to-reproductive transition the juvenile-to-adult developmental phase shift is altered, abaxial trichome development was monitored in plants growing in SDs. The number of leaves at appearance of the first abaxial trichome was recorded as well as the days until emergence of the first trichome. This showed that there was no difference in juvenile-to-adult shift between any of the genotypes (Fig. 4d). Both *ssm67* and *srr1-1* reached the adult phase a little later than wt, but the differences were not significant (Fig. 4e). This possibly reflects the slower leaf initiation rate in these genotypes compared to the wt. Thus, *ssm67* does not affect the juvenile-to-adult phase transition.

### *Hdf1-1* single mutants flower late

To isolate the novel *hdf1-1* allele from the *ssm* mutant background, *ssm67* was crossed to the Col-7 wt. Subsequently, plants without the *srr1-1* mutation were selected based on the loss of the Basta resistance of the *srr1-1* T-DNA^26^ and confirmed by PCR to carry the causal *hdf1-1* SNP.

**Figure 5:**
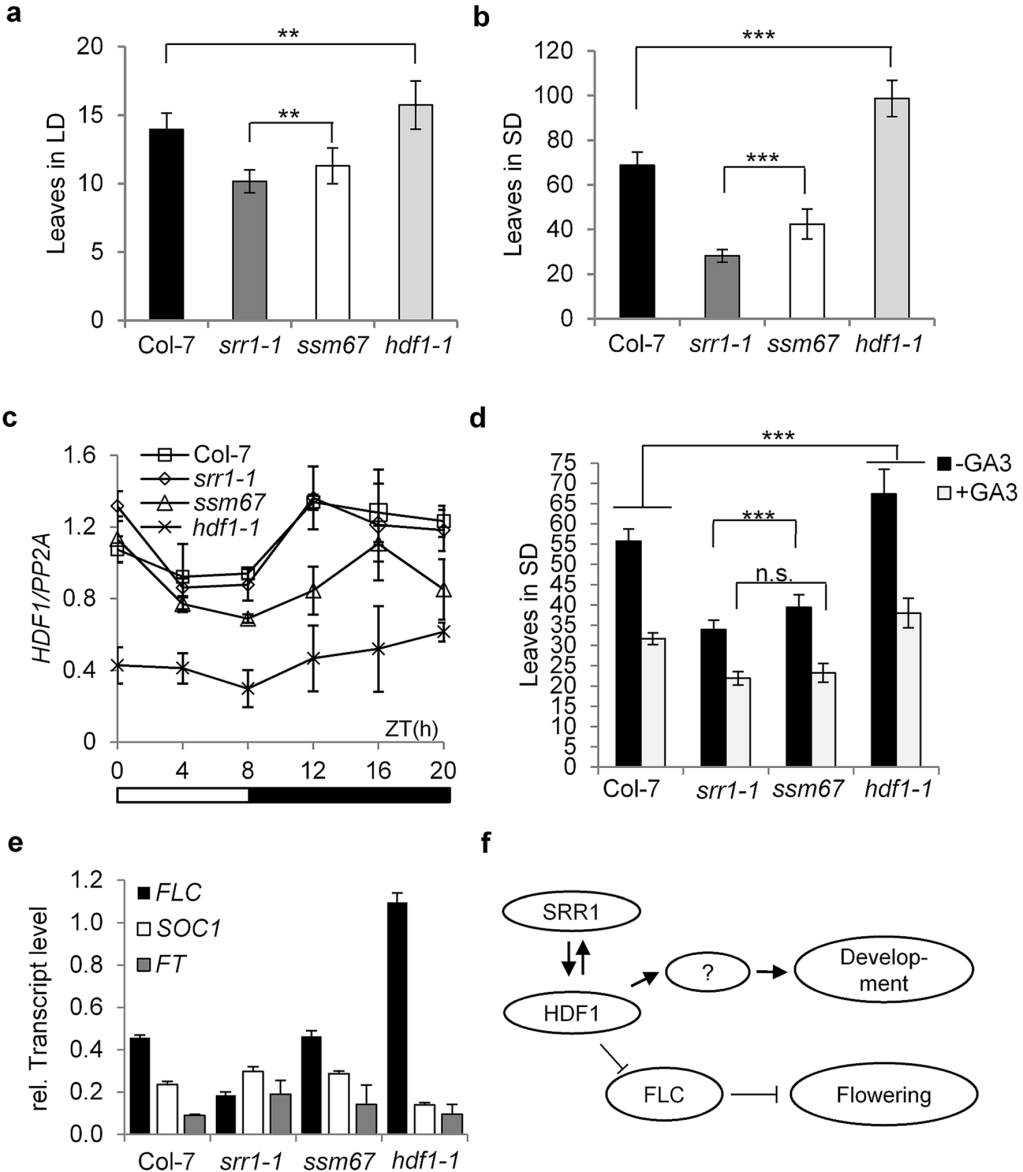
The role of HDF1 in flowering time control. Col-7 wt plants, *srr1-1*, *ssm67* and *hdf1-1* single mutants selected from a backcross of *ssm67* with Col-7 were grown in LDs (**a**) and SDs (**b**). The number of leaves was counted when the bolt was 0.5 cm in height (data are means ± s.d from n = 20–25 plants). A *t* test was employed to determine statistical significance (*P ≤ 0.05, **P ≤ 0.01, ***P ≤ 0.001). (**c**) Expression levels of *HDF1*. Col-7 wt, *srr1-1*, *ssm67* and *hdf1-1* single mutants were grown in SDs and harvested at 4-h intervals over the course of on day. *HDF1* transcript levels were monitored by RT-qPCR and normalized to *PP2A*. Data are means ± s.d. of three biological replicates. (**d**) Flowering time in response to GA. Plants were grown in SDs and sprayed with 100 µM GA_3_ or 0.1% DMSO as control (data are means ± s.d from n = 20–25 plants). A *t* test was employed to determine statistical significance (***P ≤ 0.001, *n.s*. not significant). (**e**) Expression levels of *FLC, SOC1 *and* FT*. Plants were grown in SDs for three weeks and harvested at zt4. Transcript levels were monitored by RT-qPCR and normalized to *PP2A*. Data are means ± s.d. of three biological replicates. (**f**) Proposed working model for the regulation of flowering time and vegetative development through HDF1 and SRR1. Flowering is inhibited by FLC while vegetative development could be influenced indirectly by additional unknown factors.

The single *hdf1-1* mutant plants were further characterized to examine whether the *ssm67* flowering time phenotype was dependent on SRR1. *Hdf1-1* flowers later than wt plants in LDs (Fig. 5a) and particularly in SDs (Fig. 5b). The delay of flowering in *ssm67* relative to *srr1-1* is similar to the delay of flowering in *hdf1-1* relative to Col-7, suggesting that the *hdf1-1* flowering phenotype is mostly independent of SRR1.

The *HDF1* expression profile was tested in a diurnal SD cycle in wt, *srr1-1*, *ssm67*, and *hdf1-1* background. This revealed that expression of *HDF1* is somewhat elevated during the dark period (Fig. 5c). There was no difference in expression between *srr1-1* and wt, but expression of *HDF1* was reduced in both *ssm67* and *hdf1-1.* This suggests a possible feedback regulation of the HDF1 protein on its own expression. Furthermore, *HDF1* is strongly expressed in all plant tissues according to the Arabidopsis eFP browser (Suppl. Fig. S5). This suggests that its role is not limited to floral transition.

### Impact of HDF1 on the flowering time network

In the initial screen, *ssm67* flowered much later in SDs than in LDs (Fig. 1c). Similarly, flowering of the *hdf1-1* single mutant was delayed in SDs compared to LDs (Fig. 5a,b), indicating that HDF1 is not involved in sensing day length. Therefore, we tested the effect of *hdf1-1* on flowering pathways other than the photoperiodic pathway.

To determine whether the flowering phenotype of *hdf1-1* is dependent on GA, *ssm67* and *hdf1-1* plants were tested for flowering in response to exogenous GA application. Plants grown in SDs were treated with GA every three days from 2 weeks after sowing by spraying 100 µM of GA_3_ or 0.1% DMSO as control. All plant lines reacted to the GA treatment with early flowering. The ratio of leaves with and without GA was 0.57 for wt and 0.65 for *srr1-1* indicating that either *srr1-1* reacts weaker to GA or that leaf numbers could not be reduced below a certain threshold with the applied amount of GA. *Ssm67* flowered with the same number of leaves as *srr1-1* after GA application and a ratio of 0.59, likely due to the higher initial leaf number in untreated plants. The *hdf1-1* mutant still flowered with significantly more leaves than wt after GA treatment and the ratio was 0.56, indicating that *hdf1-1* responds to GA in a similar fashion than wt (Fig. 5d). We concluded that HDF1 is not involved in GA dependent flowering, but given the weaker reaction of *srr1-1*, SRR1 might play a role in this regulatory pathway.

To examine whether the floral repressor FLC plays a role in the delayed flowering of *hdf1-1* and *ssm67* compared to *srr1-1 and* wt, *FLC* transcript levels were measured. Whereas *FLC* levels are reduced in *srr1-1*, we found a three-fold increase of *FLC* in *hdf1-1* compared to Col-7 which could explain the delay in flowering. Interestingly, despite the strong effect in *hdf1-1*, the *FLC* level in *ssm67* was only reverted back to wt suggesting the possibility that the effect of HDF1 on *FLC* is at least partly dependent on SRR1 (Fig. 5e). Although *FLC* is reverted back to wt level in *ssm67*, the plants still flower with less leaves than wt, which can be explained by the slower leaf initiation rate compared to Col-7 (Fig. 4a). In line with the observed *FLC* levels, we also measured a lower level of the floral activator *SOC1* in *hdf1-1* and a somewhat elevated level in *srr1-1* compared to wt. The expression level of *FT* was not changed in *hdf1-1*, but was elevated in *srr1-1* and *ssm67*, although with some variation (Fig. 5e). This could possibly be explained by the overall low expression of floral activators under SD conditions caused by a lack of photoperiodic induction.

We noticed that *HDF1* is located in close proximity to the *FLC* locus on chromosome five and both genes likely co-segregate. As L.*er* was used as a parent to create the mapping population from which *hdf1-1* was isolated and Col and L.*er* differ in the allelic state of *FLC*^45^ we checked whether the *FLC* allele from Col or L*.er* was present in *ssm67* and *hdf1-1*. Both lines proved homozygous for the Col *FLC* allele, thus lacking the characteristic 1.2 kb insertion indicative of the weak L.*er* allele (Suppl. Fig. S6a,b). Therefore, we could rule out differences in the allelic state of *FLC* as a cause for the observed differences in *FLC* abundance. Taken together, our data suggest that *FLC* could be a major cause for the reverted flowering phenotype of *ssm67.* This leads us to a model where SRR1 impacts flowering time via HDF1 through the regulation of *FLC* and additionally affects plant development during the vegetative phase also through HDF1, but possibly also involving other factors. Whether there is a reciprocal interaction of SRR1 and HDF1 has yet to be determined (Fig. 5f).

## Discussion

In a search for novel genes important for the regulation of flowering by SRR1, we embarked on a second-site suppressor screen for mutants that rescue the *srr1-1* early-flowering phenotype under noninductive SD conditions. As the loss of SRR1 and PhyB additively accelerate flowering time at higher temperatures, the screen was performed at 16 °C, where PhyB has no influence on flowering time^25^. Candidate mutants were then rescreened at 20 °C. This screening scheme allowed for identification of flowering suppressors in an early-flowering background reducing the risk of overseeing mutant candidates whose increase in leaf number would be difficult to quantify in wt background due to leaf senescence of earlier developed leaves. The screen identified a range of mutant candidates with a flowering phenotype intermediate between *srr1-1* and Col-7 (Fig. 1a).

To identify the position of the causal mutation of *ssm67*, a combination of linkage mapping and full genome sequencing was used. The dominant segregation pattern of the mutation in *ssm67* hindered the enrichment of homozygous candidates for the bulk-segregant analysis, leading to a distribution of potential SNP candidates in the sequencing data on three chromosomes, with no candidate SNP reaching a ΔSNP index over − 0.8 (Fig. 2a). The linkage mapping, however, revealed that a region on the top of chromosome V harbors the causative SNP (Fig. 2b), narrowing down the number of candidate loci to three (Suppl. Table S2). The SNP in position 683 of *HDF1* could then be confirmed as the causal mutation by genotyping an independent segregation BC1F2 population, revealing that its presence was necessary for the late flowering phenotype (Fig. 2d). The complementation of *ssm67* with the genomic copy of the *HDF1* gene restored the *srr1-1* flowering phenotype, further confirming that the mutation in *HDF1* is responsible for the delay in flowering (Fig. 2e). Our results show the strength of combining a classical linkage mapping and state-of-the-art full genome resequencing when the mutated population does not display a purely Mendelian segregation pattern. In this way, we were able to identify a novel flowering time regulator from a dominantly segregating population, which masked its position in the sequencing data.

The novel flowering time regulator we identified turned out to be a HAD superfamily protein. These proteins are characterized by a core catalytic domain that is known as a Rossmannoid fold and is comprised of four diagnostic motifs^33^. The mutant allele we identified in our screen affects none of these motifs directly, but is instead located in the intervening region between motif III and IV (Fig. 3c). The Ala to Val exchange is considered a conservative replacement, as both are aliphatic non-polar amino acids. This raises the possibility that the observed effects in the *ssm67* and *hdf1-1* plants are rather mild. In the future, the CRISPR/Cas technology can be used to engineer a complete loss-of-function allele both in the wt background and the *srr1-1* background. This may provide more insights into the degree of genetic interaction between HDF1 and SRR1 in flowering time control and development.

The HAD superfamily is by far dominated by ATPases and acid-phosphatases^46^, but information on substrates cannot be gained from sequence comparison alone but instead has to be determined experimentally. So far, there is only minor evidence on what as possible substrate could be. The presence of a large C2 cap between the conserved motifs II and III, restricting access to the active site, points to HDF1 acting on a small molecular rather than on a larger substrate. HDF1 was found previously to be related to AtFHy1 encoding an FMN hydrolase. AtFHy1 indeed was shown to dephosphorylate the riboflavin biosynthesis pathway intermediate 5-amino-6-ribitylamino-2,4(1H,3H) pyrimidinedione 5′-phosphate (ARPP) in vitro whereas HDF1 was not active on this substrate^43^.

The close relationship to HAD type phosphatases may suggest that HDF1 also acts as a phosphatase. The absence of the diagnostic second Asp in motif I, however, could also suggest that HDF1 may act as a haloacid dehalogenase, as found in bacteria. Additionally, the possibility that HDF1 acts on multiple substrates cannot be ruled out, as HADs with a C2 cap were found to be less substrate specific than HADs with a C1 or a C0 cap^47^.

It is likely that HDF1 plays a more general role in plant metabolism than solely being limited to floral transition. This may be reflected by the developmental phenotypes we found in *ssm67*. The rosette size of *ssm67* mutants was strongly reduced and the leaf emergence rate was accelerated in *ssm67* compared to *srr1-1* (Fig. 4c–e). The widespread expression throughout development (Arabidopsis eFP browser) as well as the high level of conservation in the *Brassicaceae* also point in this direction (Suppl. Fig. S4). This is further strengthened by a recent study that identified 41 *HAD* genes in rice and 40 *HAD* genes in Arabidopsis. 17 of the HADs from rice were induced upon phosphate starvation and thus were predicted to be involved in phosphate recycling under Pi stress conditions^37^. As the search was limited to HADs that show a conserved DxD pattern in motif I, HDF1 was omitted from the search.

*HDF1* expression shows a weak diurnal fluctuation, with an elevated level in the dark period (Fig. 5c). Notably, *HDF1* expression was reduced in both *ssm67* and *hdf1-1*, pointing to a positive feedback regulation that might be disturbed by the Ala to Val exchange. As such, it is likely that the mutation we identified creates a hypomorphic allele. *HDF1* expression was previously found to be reduced in *phyA* and *phyB* mutants, placing red light as a putative upstream regulator of HDF1^48^. However, as *HDF1* expression was not strongly altered in *srr1-1*, the effect of PhyB may be independent of signaling through SRR1.

To define how HDF1 acts in the network of flowering time regulators, we tested the involvement in several known pathways. *Ssm67* and *hdf1-1* were late flowering in SDs as well as in LDs, indicating that the photoperiodic response is intact and that HDF1 is not involved in sensing day length (Fig. 5a,b). Furthermore, *ssm67* flowered with more leaves than *srr1-1* both at 16 °C and 20 °C, suggesting that HDF1 does not affect the temperature response (Fig. 1a,b).

When plants grown in SDs were treated with GA, the transition to flowering was accelerated in *hdf1-1* and *ssm67* similarly as in wt, ruling out also this prominent pathway of flowering time regulation. Notably, the response of *srr1-1* to GA was somewhat reduced. This may indicate that either early flowering could not be accelerated further, or that *srr1-1* reacts weaker to GA (Fig. 5d).

Ultimately, we monitored *FLC* levels to address a role in the autonomous regulation of flowering time. We found *FLC* to be strongly increased in *hdf1-1*. Interestingly, despite the strong effect in *hdf1-1*, the *FLC* level in *ssm67* was only reverted back to wt. (Fig. 5e). The *FLC* allele from the Landsberg ecotype carries a 1.2 kb insertion of a *Mutator*-like transposon in intron 1 causing low *FLC* steady-state abundance^45^. To rule out that the differences in *FLC* levels of *hdf1-1* and *ssm67* are a consequence of segregating *FLC* alleles either from the Landsberg background used to generate the mapping population or the Col background used for backcrossing, we genotyped the *FLC* allele. We found that in *srr1-1*, *hdf1-1* and *ssm67* the *FLC* allele from Col was present in a homozygous state (Suppl. Fig. S6). This suggests the possibility that functional SRR1 is needed for proper HDF1 function on *FLC* and that the differences in *FLC* expression between *ssm67* and *hdf1-1* are not caused by *FLC* alleles from different genetic backgrounds. But, as *hdf1-1* is late-flowering compared to Col-7 wt in both LD and SD conditions, it is obvious that besides this partial dependency on SRR1, HDF1 influences flowering also independently of SRR1. FLC is a strong repressor of flowering, but its roles are not limited to flowering time pathways. Instead, among its numerous targets are many other transcripts implicated in the regulation of developmental phase transition, i.e. hormonal response genes, floral meristem identity genes, or the transcription factor SPL15 involved in the juvenile to adult phase transition^49,50^. This could mean that changes in the FLC level might be responsible for other developmental phenotypes of *srr1-1*.

To our knowledge, this is the first time that a HAD superfamily protein has been implicated in flowering time control in Arabidopsis. Previously, a protein phosphatase from tomato, LePS2;1, has been shown to be induced upon phosphate starvation, and overexpression in transgenic plants led to increased anthocyanin accumulation, changes in morphology and delayed flowering^51^. LePS2;1 dephosphorylates synthetic phosphor-Serine/Threonine peptides in vitro. It differs from HDF1 in that overexpression rather than loss-of-function delays flowering.

In addition to the *ssm67* mutation, we characterized the *ssm15* mutation which also flowers intermediate between *srr1-1* and wt. Full genome sequencing led to the identification of a G to A transition in exon 6 of ARABIDOPSIS SUMO PROTEASE 1 (ASP1), leading to an Ala to Thr exchange (Suppl. Fig. S2c). ASP1 was recently shown to positively regulate the transition to flowering at least partly by repressing FLC protein stability^31^. *FLC* displayed wt levels in *ssm15* (data not shown), The fact that we identified two regulators of FLC with very distinct functions may be a hint that FLC could be a determinant of SRR1 mediated flowering and further emphasizes the role of this repressor as a major hub of flowering time control in general.

In conclusion, our large scale genetic screen identified candidates that suppress the early flowering phenotype of *srr1-1*. On closer inspection, we could not only find flowering related phenotypes, but were able to describe a potential role of SRR1 in vegetative development for the first time. The leaf emergence rate in *srr1-1* was significantly lower than in wt and this was in large parts rescued in the *ssm67* mutant. This indicates that the developmental phenotype is at least partly dependent of HDF1. In contrast, *ssm67* partly rescued the early flowering phenotype of *srr1-1*, but *hdf1-1* also delayed flowering in wt. This might indicate that the effect on flowering time is regulated independently of SRR1. *FLC* was identified as a major conversion point in the suppressor candidates *ssm15* and *ssm67*, suggesting that ASP1 and HDF1 bypass the requirement for SRR1 to downregulate floral repressors in inductive conditions by the strong repression of *FLC*.

## Materials and methods

### Plant material, growth conditions and suppressor screen

All Arabidopsis plant lines used in this study are derived from common laboratory strains. No seeds or plant material was collected from the wild and all methods involving plants were carried out according to institutional, national, and international guidelines and legislation.

The T-DNA mutant *srr1-1* in the Col-7 background has been described^26,27^. All seeds were stratified for 3 days at 4 °C before grown on soil. Seeds were surface sterilized and stratified for 3 days at 4 °C before plating on agar-solidified half-strength MS (Murashige and Skoog) medium (Duchefa) supplemented with 0.5% sucrose and 0.5 g MES. Plants were grown in AR66-L3 Percival incubators (CLF Laboratories) in 100 µmol m^−2^ s^−1^ light intensity, with the light–dark and temperature conditions as indicated.

For the second-site suppressor screen, ca 20.000 *srr1-1* seeds were mutagenized overnight in sodium phosphate buffer (pH 7.5)^52^. Subsequently, 0.3% ethylmethanesulfonate (Sigma Aldrich) was added and the mixture was incubated for 15 h with rotation. Seeds were then washed 20 × with water and distributed on soil in 280 batches. After 3 days of stratification at 4 °C, the seeds were transferred to LD growth conditions. M1 seeds were harvested in batches and 100 M2 seeds each from 11 randomly selected batches were used for the initial screen.

### Flowering time analysis

Seeds were germinated as described above and grown on soil in a random fashion. Flowering time was determined by counting the rosette leaves when the bolt was > 0.5 cm tall^53^. For GA treatment, plants growing on soil were sprayed with 100 µM GA_3_ four to six h after lights on twice a week starting at day 14 after stratification. Mock treatment was performed by spraying with 0.1% DMSO/0.02% Tween 20.

### Determination of leaf initiation rate and rosette size

Leaf initiation rate was scored by counting the number of rosette leaves every 3 days starting 10 days after sowing and plotting the number of leaves against days of growth. Counting was done until the plant started to flower.

Rosette size of the plants was monitored by taking pictures at the indicated time points starting 28 days after sowing. The area covered by the rosette was determined using the software Rosette tracker^54^.

### Abaxial trichome analysis

As a measure of the juvenile-to-adult developmental transition, plants were scored for the presence of abaxial trichomes every 3 days starting 10 days after sowing. When an abaxial trichome was identified, the leaf number was notified and the plant was considered as adult and no longer scored.

### DNA extraction for sequencing

Leaf material was sampled from 35 individuals in segregating *ssm67* × *srr1-1* BC1F2 and *ssm15* × *srr1-1* BC1F2 population consisting of 115 plants each. Plants with at least 25% more leaves at bolting compared to *srr1-1* control plants were selected, the material was frozen in liquid N_2_ and ground to a fine powder. Equal amounts of each plant sample were pooled and this pool was extracted using a DNeasy Plant Maxi Kit (Qiagen) according to the manufacturer’s instructions, with the following exceptions: 2 × the amount of lysis buffer was used, all centrifugation steps were done for 10 min, and the empty column was centrifuged for 15 min and heated at 37 °C for 10 min before elution to avoid ethanol contamination.

### Sequencing

Four µg of DNA from the *ssm15* and *ssm67* sequencing populations was sent to Novogene (Hong Kong) for sequencing and mapping to the reference genome. After quality control, libraries were constructed using the Illumina TruSeq Library Construction Kit. Pair-end sequencing were performed on Illumina^®^ HiSeq platform, with the read length of PE150 bp at each end. Base calling was done with the CASAVA software. Mapping was performed by Novogene using the BWA, SAMtools and Picard softwares, followed by SNP/Indel detection (SAMtools) and variation annotation (ANNOVAR). The same process was used to sequence a pool of *srr1-1* plants. SNPs were determined relative to the TAIR10 reference as of May 23rd, 2017.

To create a SNP candidate index and avoid false detection of polymorphisms, all SNPs also present in *srr1-1* were filtered out, as well as low-quality and multiple-hit reads^55^. The SNP index was calculated by comparing the total number of reads to the number of reads for a non-reference base and plotted according to position on the chromosome.

### Linkage mapping

To narrow down the number of candidates, a linkage mapping population was created by crossing the *ssm67* mutant with the Landsberg *erecta* wt. Rough mapping was performed in the resulting segregating population by testing two markers per chromosome using PCR^56,57^. The region where candidate loci from the whole-genome sequencing approach were located were examined in more detail using additional markers. PCR was used to amplify nucleotide sequences from simple sequence length polymorphisms (SSLP) or cleaved amplified polymorphic sequences (CAPS) markers. In the case of the CAPS markers, the PCR product was digested with restriction enzymes according to the manufacturer’s instructions. The products were subsequently separated on an agarose gel and analyzed.

### dCAPS primer design

To determine the presence of SNPs in the mutagenized plants, primers were designed to amplify the genomic region surrounding the SNP of interest. This was followed by digestion of the PCR product by restriction enzymes that only digested either the mutated product or the wt product, according to the Derived Cleaved Amplified Polymorphic Sequences (dCAPS) method. Primers were designed using the dCAPS finder software, 1–2 mismatches were added to the primer to create specific restriction sites, allowing digestion of only mutant or only wt sequence^58^. The sequence of the primers are listed in Suppl. Table S1.

### Complementation of the *ssm67* mutant

1.5 kb of the promoter and the genomic sequence of At5g10460 was amplified with primers adding BamHI restriction sites and cloned into the pJET1.2 cloning vector (Thermo Scientific). The fragment was cut and introduced into the binary vector pCAMBIA3300 using BamHI and transformed into *Agrobacterium tumefaciens* GV3101. Plants were transformed by floral dip^59^.

### Transcript analysis

Total RNA was extracted from plant material using Universal RNA Purification Kit (Roboklon) following the manufacturer’s instructions. For cDNA synthesis, 2 µg of total RNA was DNase-treated using RQ1 RNase-free DNase (Promega) and reverse transcribed using AMV Reverse Transcriptase (Roboklon) according to the manufacturer’s instructions. qPCR was performed with iTaq Sybr Green Supermix (Bio-Rad) according to manufacturer’s instructions. The normalized expression level was determined using the ΔCt method, with *PP2a* (At1g13320) as a reference gene as described^60^. To amplify *FT* in SD grown samples, a nested PCR was performed. The primer sequences can be found in Suppl. Table S1.

### Construction of the phylogenetic tree

To construct the phylogenetic tree of HAD family proteins, a dataset was compiled comprised of 21 genes from (dataset 1)^35^, 55 genes from (dataset 2)^37^, and 58 proteins which were identified as haloacid dehalogenase by textmining Araport 11 genes (dataset 3). Overall, 93 unique genes from the three datasets were used for further analysis.

The protein sequences were downloaded via the *thalemine* web interface (https://bar.utoronto.ca/thalemine/begin.do (accessed August 2021)). These sequences were aligned using the MAFFT version 7 online Alignment server (https://mafft.cbrc.jp/alignment/server/) with the ‘G-INS-i’ parameter for sequences with global homology and the BLOSUM62 scoring matrix for amino acids^61,62^.

The resulting full-length sequence alignment was translated into the PHYLIP format and uploaded to the PHYML 3.0 web server (http://www.atgc-montpellier.fr/phyml/). The detection of the optimal substitution model was set to automatic model selection SMS^63^ with the Akaike Information Criterion (AIC) parameter. The tree was constructed using the fast likelihood-based method ‘aLRT SH-like’. The SMS of the first tree determined ‘WAG + G + I + F’ to be the best fitting model. The resulting tree was visualized using the PHYML 3.0 online resource PRESTO (http://www.atgc-montpellier.fr/presto/).

For construction of the phylogenetic tree of the *HDF1* orthologs in other *Brassicaceae*, protein sequences from selected organisms were obtained from the UniProt database (http://www.uniprot.org). The tree was constructed using the same tools as outlined above, with the exception that the automatic model selection SMS determined ‘JTT + G’ to be the best fitting model.

## Supplementary Information

Supplementary Information.

## Data Availability

Data supporting the findings of this work are available within the paper and its Supplementary Information file. Sequencing data generated for *ssm15* are accessible at NCBI’s Sequence Read Archive (SRA) via accession number [SRR22318065]. Sequencing data generated for *ssm67* are accessible at SRA via accession number [SRR22318066].
