# Supplementary material for: HDF1, a novel flowering time regulator identified in a mutant suppressing sensitivity to red light reduced 1 early flowering
Source: Sci Rep. 2023 Jan 25;13:1404. doi: 10.1038/s41598-023-28049-6 (PMC9876914; doi:10.1038/s41598-023-28049-6)
Supplement: Supplementary file 1 — Supplementary Information. [file 41598_2023_28049_MOESM1_ESM.pdf]

HDF1, a novel flowering time regulator identified in a mutant suppressing *sensitivity to red light reduced 1* early flowering

Mikael Johansson[1+,*], Alexander Steffen[1+], Martin Lewinski[1], Natalie Kobi[1], and Dorothee Staiger[1]*

[1]RNA Biology and Molecular Physiology, Bielefeld University, Universitaetsstrasse 25, 33615 Bielefeld, Germany

***** dorothee.staiger@uni-bielefeld.de; mikael.json@gmail.com

[+] joint first authors

Supplementary material

Suppl. Fig. S1

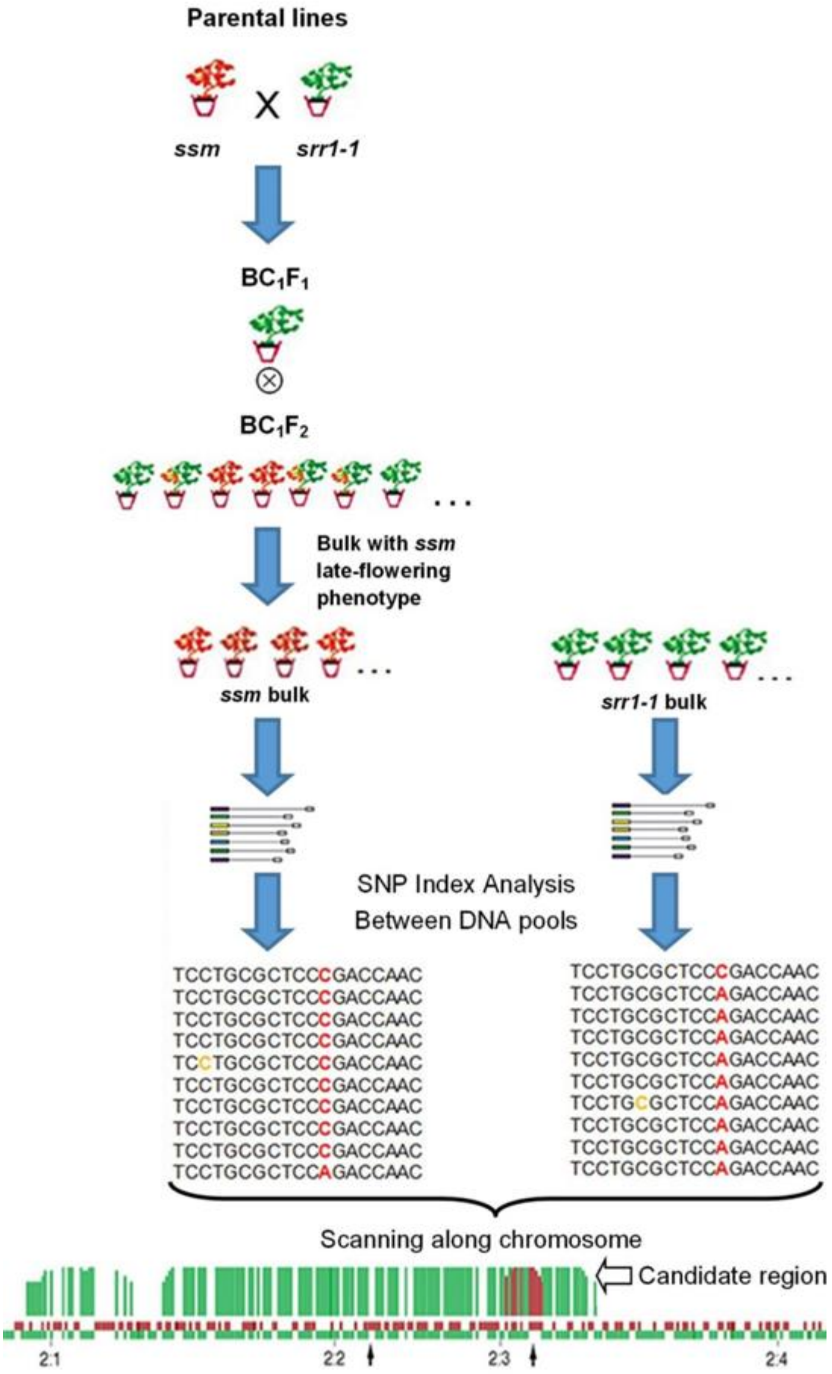

Supplementary Figure S1. Design of the full genome re-sequencing of the *ssm* mutations The *ssm* lines were backcrossed to *srr1-1* and the resulting BC1F1 populaton was selfed. In the segregating BC1F2 population plants displaying the late flowering *ssm* phenotype were bulked for DNA isolation and full genome sequencing. A bulked *srr1-1* population was sequenced accordingly as a reference. A SNP index for all mutations was calculated for both pools to identify SNPs unique for the *ssm* mutants.

Suppl. Fig. S2

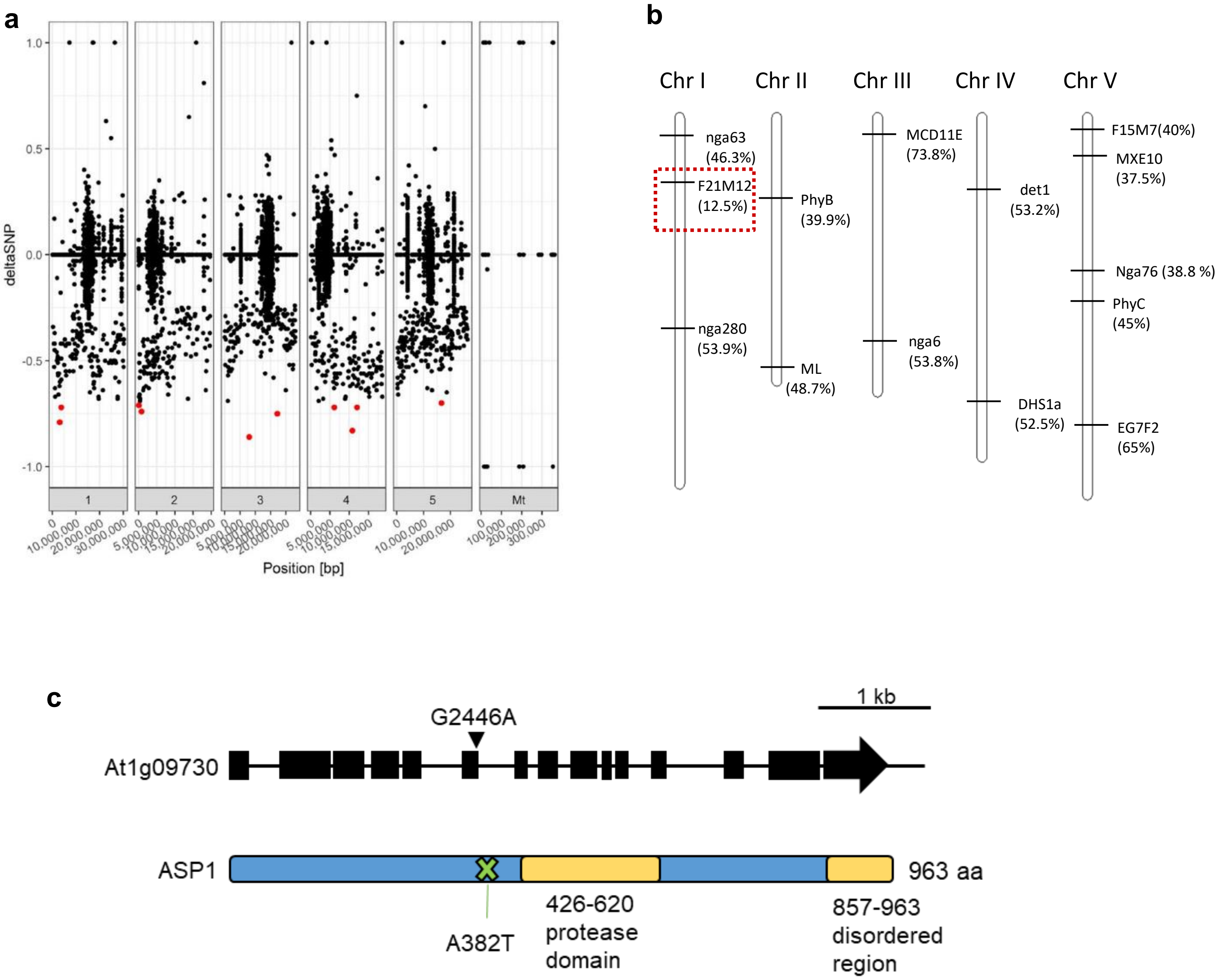

Supplementary Figure S2. Identification of the causal mutation in *ssm15*
(a) Distribution of all identified SNPs in the *ssm15* genome and their ΔSNP index compared to the *srr1-1* genome. A ΔSNP index closer to -1.0 indicates SNPs unique for *ssm15*. The red dots denote SNPs ≤ -0.7, all SNPs below this value were evaluated as potential causal SNPs.
(b) Linkage mapping of the causal mutation in a *ssm15* x L. *erecta* mapping population using SSLP and CAPS markers.
(c) Scheme of *ASP1* and the resulting protein. Black boxes represent exons. The SNP in *ASP1* and the resulting Ala to Thr exchange is indicated.

Suppl. Fig. S3

a

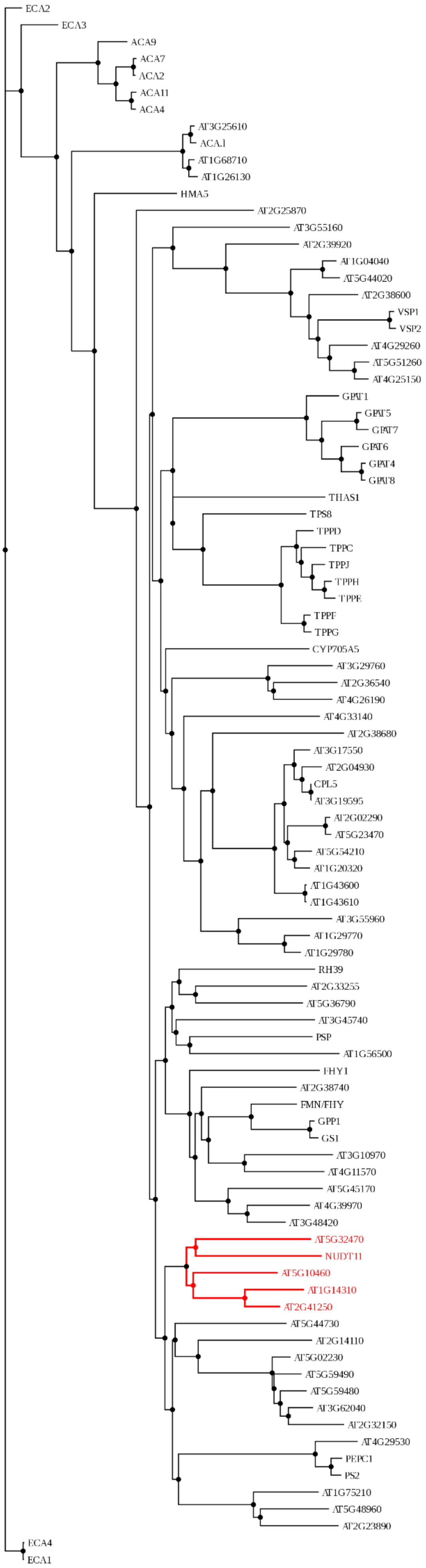

b

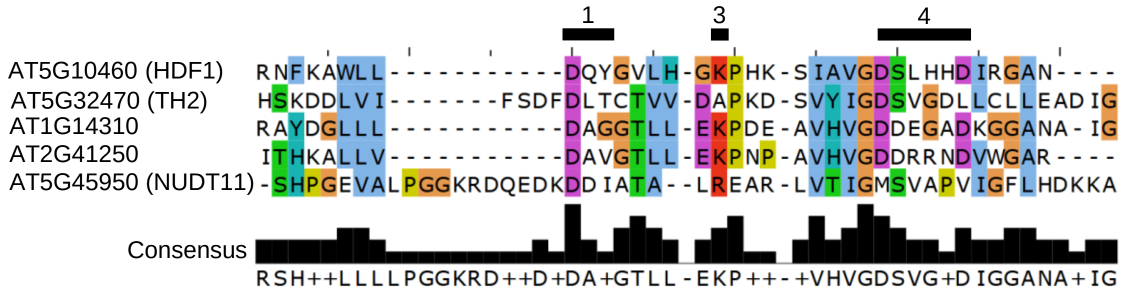

Supplementary Fig. S3. Approximation of a phylogenetic tree of 93 HADs in Arabidopsis
(a)Phylogenetic tree of 93 HAD superfamily proteins from Arabidopsis. Alignment of the predicted sequences was made using MAFFT version 7. The phylogenetic tree was constructed using the PHYML 3.0 web server. The sub-clade containing HDF1 is highlighted in red.
(b) Alignment of the core diagnostic motifs 1, 3 and 4 of the sub-clade containing HDF1, AT5G32470 (TH2), AT1G14310, AT2G41250 and AT5G45940 (NUDT11). Motif 2 appears not conserved. A consensus sequence is given below.

Suppl. Fig. S4

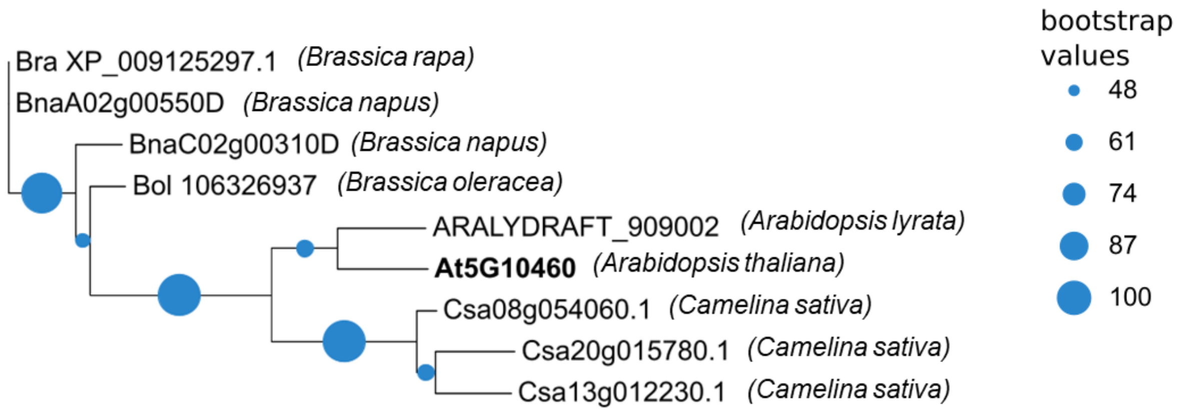

Supplementary Fig. S4. HDF1 orthologues in other members of the *Brassicaceae* Phylogenetic tree of HDF1 orthologous sequences from *Arabidopsis lyrata, Camelina sativa, Brassica oleracea, Brassica rapa*, and *Brassica napus*. Sequences were obtained from the UniProt database (www.uniprot.org). Alignment of the predicted sequences was made using MAFFT version 7. The phylogenetic tree was constructed using the PHYML 3.0 web server.

Suppl. Fig. S5

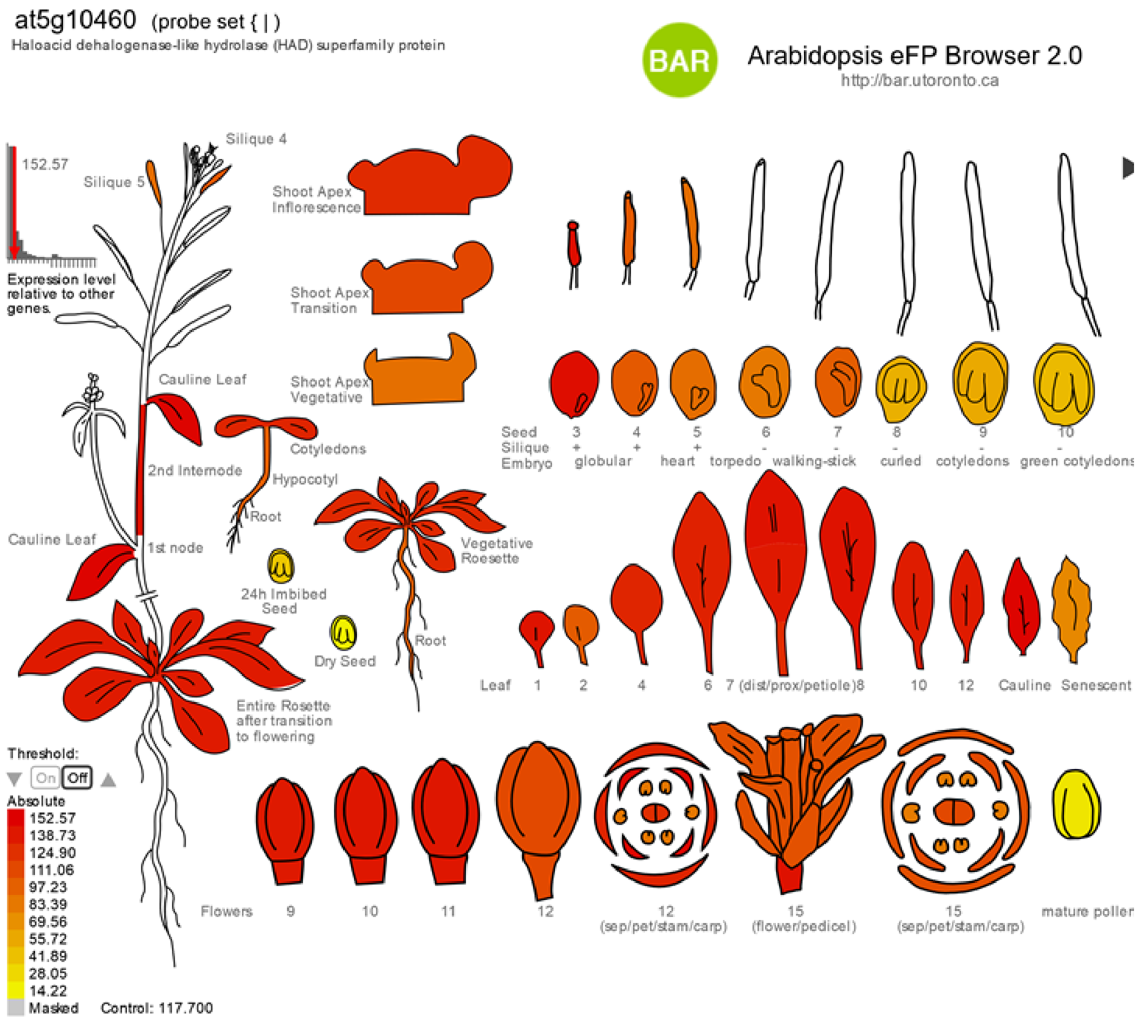

Supplementary Figure S5. Expression of *HDF1*
Expression level of *HDF1* in various tissues according to the Arabidopsis eFP browser (http://bar.utoronto.ca/efp2/).

Suppl. Fig. S6

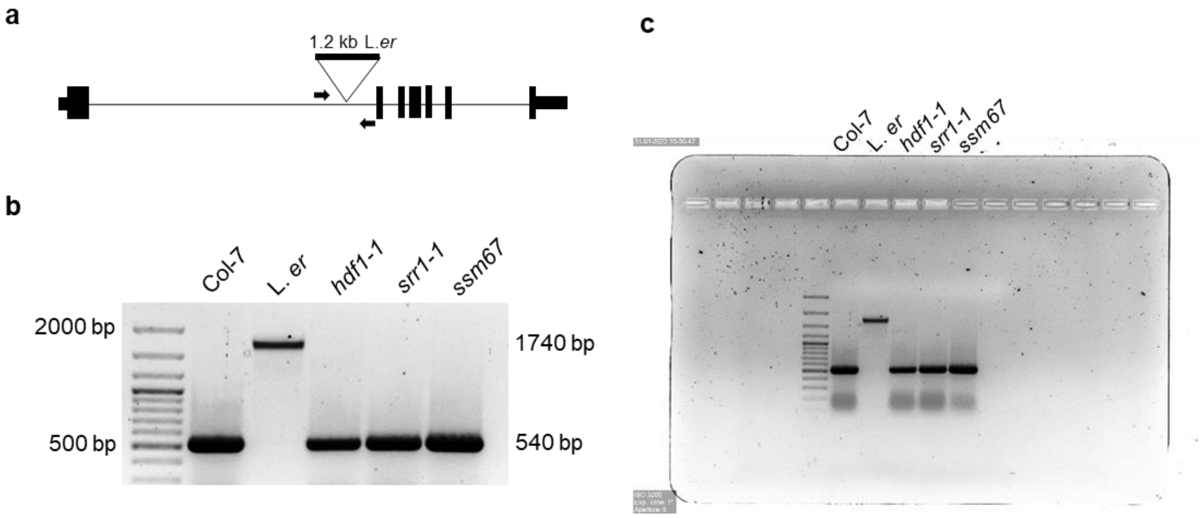

Supplementary Figure S6. Identification of the *FLC* allelic state

(a) Schematic representation of the *FLC* locus. Large boxes represent exons, thin boxes represent UTRs. Introns are represented as lines. The triangle denotes the location of the 1.2 kb insertion in intron 1 of L. *er*. Primers used to amplify the region are denoted as arrows. (b) Genomic DNA was isolated from Col-7, L.*er*, *hdf1-1*, *srr1-1* and *ssm67* and amplified with primers shown in (a). Fragments were separated on an agarose gel. (c) Uncropped gel image.

Supplementary Table S1: Oligonucleotides used in this study

Oligonucleotide	AGI	Symbol	Sequence	Properties
nga 63 for		Marker	ACCCAAGTGATCGCCACC	SSLP, Col 111 bp; L. er 89 bp
nga 63 rev		Marker	AACCAAGGCACAGAAGCG	
nga280 for		Marker	GGCTCCATAAAAAGTGCACC	SSLP, Col 174 bp; L. er 132 bp
nga280 rev		Marker	CTGATCTCACGGACAATAGTGC	
F20P5 for		Marker	TGCCTTGTTCATCGTCTC	SSLP, Col 175 bp; L. er 124 bp
F20P5 rev		Marker	TGGGCGTCTTGTCGATGT	
F21M12 for		Marker	TGCTTCCTAAGTTCATCAT	SSLP, Col 155 bp; L. er 134
F21M12 rev		Marker	TGTAAACCAAGAATCCAAA	
PhyB for		Marker	CAATCCTATGAAGAATGGCG	CAPS XhoI cuts L. er 0.7+0.4 kb
PhyB rev		Marker	ATAAACCATTAGCCCACGTG	
VE017for		Marker	GAGCAATCCAGTAGAGGATA	CAPS PstI cuts Col 214+143 bp
VE017rev		Marker	CTTGAAGCTTAAATCTCAGC	
MLfor		Marker	CGGAAACACGAAGCTGATGAGTTGGG	CAPS SnaBI cuts Col 0.5+0.4 kb
MLrev		Marker	CGAGAACAAAATGTGTACGGTGTG	
MDC11Efor		Marker	GATAGATCTTCGTGAGTCGTCTGT	CAPS XhoI cuts L. er 0.75+0.5 kb
MDC11Erev		Marker	GCATCGTGCTTGGTTGCTCGTGCA	
nga6for		Marker	ATGGAGAAGCTTACACTGATC	Col 143 bp; L. er 123 bp
nga6rev		Marker	TGGATTTCTTCCTCTCTTCAC	
det1for		Marker	GAGCATCAACAAGATGACC	CAPS SacI cuts L. er 0.6+0.5 kb
det1rev		Marker	CAAAATGTGAATGTCC	
DHS1afor		Marker	AGAGAGAATGAGAAATGGAGG	CAPS DdeI cuts Col 1491+129+48 bp; cuts L.er 1620+48 bp
DHS1arev		Marker	CAAGTGACCTGAAGAGTATCG	
F15M7for		Marker	CTCATGCTTCATCAAGTTCT	SSLP, Col 92 bp; L.er 98 bp
F15M7rev		Marker	CATGATGCTAGGAAATGTTA	
MXE10for		Marker	CTCCCTGGTAATGTTAATCC	SSLP, Col128bp; L.er 116 bp
MXE10rev		Marker	CCTGCACATTGATCACCA	
nga76for		Marker	FAGGCATGGGAGACATTTACG	SSLP, Col 231 bp; L.er 0.3+0.4 kb
nga76rev		Marker	GGAGAAAATGTCACTCTCCACC	
Phy Cfor		Marker	CTACAGAATCGTCCTCAACG	CAPS PstI cuts L.er 0.8+0.7 kb
Phy Crev		Marker	CCTAATGGAGAATCATTCGG	
EG7F2for		Marker	GCATAGAATTTGACGATAACGAGC	CAPS XbaI cuts L.er 0.7+0.5 kb
EG7F2rev		Marker	GATCTGTGTAGGACTACGAGAC	
dCAPS_ssm67F	At5g10460	*HDF1*	ATCCAGATTACGTGACTGTT	dCAPS NcoI cuts wt 120+33bp
dCAPS_ssm67R	At5g10460	*HDF1*	GACTCAGACGGATTGACTCCCGCAATTGCCATG	
dCAPS_ssm15F	At1g09730	*ASP1*	GCTTACTCGAGGTCAGTATTCCACAGAG	dCAPS HinfI cuts mutant 101+28bp
dCAPS_ssm15R	At1g09730	*ASP1*	CATGTGGTTTCCAGACATGG	
genHADF	At5g10460	*HDF1*	CGCGGATCCGTTTGGGGTTTGTGATAAGA	Cloning HDF1 genomic fragment
genHADR	At5g10460	*HDF1*	CGCGGATCCCTTTCTCAGCAAATGAGCTT	
FLC_295	At5g10140	*FLC*	CTTGTGGATAGCAAGCTTGTGGG	RT-qPCR
FLC_417	At5g10140	*FLC*	CATGAGAGTTCGGTCTTCTTGGCTC	
HDF1-qpcr_for	At5g10460	*HDF1*	ATCAATCTTTGCAGAGGAGA	RT-qPCR
HDF1-qpcr_rev	At5g10460	*HDF1*	CGCTAAAACAAAGTCAGCTT	
PP2A_for	At1g13320	*PP2A*	CGATAGTCGACCAAGCGGTT	RT-qPCR
PP2A_rev	At1g13320	*PP2A*	TACCGAACATCAACATCTGG	
SOC1_415	At2g45660	*SOC1*	CGAGCAAGAAAGACTCAAGTGTTTAAGG	RT-qPCR
SOC1_525	At2g45660	*SOC1*	TTCATGAGATCCCCACTTTTCAGAGAG	
FT_243	At1g65480	*FT*	CCTCCGAGAATATCTCCATTG	RT-qPCR

FT_364	At1g65480	*FT*	ACACGACACGATGAATTCCTG	
FT_336	At1g65480	*FT*	GCCAAAGGTTGTTCCAGTTGTAG	RT-qPCR
FT_381	At1g65480	*FT*	GGAACTGGCTCACAAGTCCAA	
FLCint1_genot_f	At5g10140	*FLC*	AAACAATCTGGACAGTAGAGGCTTAT	Genotyping FLC
FLCint1_genot_r	At5g10140	*FLC*	CAGGCTGGAGAGATGACAAAA	

Supplementary Table S2: Candidate SNPs for the *ssm67* mutation on chromosome 5

ΔSNP	Type	Chr	Pos	Ref	Alt	AGI	Gene info
-0.77	exonic - nonsynonymous	5	3289606	C	T	AT5G10460	Haloacid dehalogenase-like hydrolase (HAD) superfamily protein
-0.76	exonic - nonsynonymous	5	7743521	C	T	AT5G23080	TGH RNA binding protein
-0.72	exonic - nonsynonymous	5	19946960	C	T	AT5G09670	loricrin-like protein

ΔSNP: Score of the SNP relative to srr1-1 and the Col reference genome

Type: location and consequence of the amino acid exchange

Chr: Chromosome

Pos: Position on the chromosome

Ref:nucleotide in the reference sequence

Alt: nucleotide in the mutant sequence
